# The role of welfare regimes in the relationship between childhood economic stress and adult health: a multilevel study of 20 European countries

**DOI:** 10.1016/j.ssmph.2020.100674

**Published:** 2020-09-30

**Authors:** Tarjei Widding-Havneraas, Siri Hansen Pedersen

**Affiliations:** a Centre for Research and Education in Forensic Psychiatry, Haukeland University Hospital, Bergen, Norway; b Department of Clinical Medicine, University of Bergen, Norway; c Department of Comparative Politics, University of Bergen, Norway

**Keywords:** Self-rated health, Cancer, Life course, Welfare regime, Social determinants, Multilevel

## Abstract

Childhood economic conditions are important for adult health, and welfare regimes may modify this relationship by altering exposure to social determinants of health. We examine the association between childhood economic stress (CES) and self-rated health (SRH) and cancer (any type), and how welfare regimes may influence these associations. We used data from European Social Survey round 7. Our study is based on 30 024 individuals between 25 to 75 years from 20 European countries grouped into five welfare regimes (Scandinavian, Anglo-Saxon, Bismarckian, Southern and Eastern). Multilevel models were used to assess the association between CES and SRH/cancer, and interactions between CES and welfare regimes. CES increased the risk of poor SRH (RR 1.41, 95% CI 1.29–1.54) and cancer (RR 1.19, 95% CI 1.02–1.37). Controlling for adult socioeconomic status slightly reduced risk for poor SRH, but not cancer. CES increased the probability of poor SRH in the Southern and Eastern regime, and the probability of cancer in the Anglo-Saxon regime, relative to the Scandinavian regime. Childhood economic stress increases the risk of poor self-rated health and cancer. More comprehensive welfare states mitigate these associations, which emphasizes the impact of welfare policies on long-term health outcomes of childhood economic conditions.

## Highlights

• The association between childhood economic stress and adult self-rated health and cancer was examined.
• The interaction of childhood economic stress and welfare regimes was tested on a large data set with 20 European countries.
• The article draws on welfare regime and life course theory.
• Childhood economic stress increased risk of poor self-rated health and cancer in adulthood.
• Welfare regimes affected the association between childhood economic stress and adult poor self-rated health and cancer.

## 1 Introduction

Social conditions and exposures shape our health throughout our whole life course (Berkman, Kawachi, & Glymour, 2014; Krieger, 2011). Social inequalities in our childhoods often transfer to health inequalities later in life (Cohen, Janicki-Deverts, Chen, & Matthews, 2010; Glymour, Avenado, & Kawachi, 2014). Welfare states follow individuals from the cradle to the grave and are considered vital to public health and health inequalities. Lower childhood socioeconomic conditions are associated with several adverse health outcomes, including higher mortality (Cohen et al., 2010; Galobardes, Lynch, & Smith, 2008), higher risk of cardiovascular diseases (Su, Jimenez, Roberts, & Loucks, 2015), cancer (Akinyemiju et al., 2018; de Kok et al., 2008; Vohra, Marmot, Bauld, & Hiatt, 2016), common mental disorders (Bøe, Balaj, Eikemo, McNamara, & Solheim, 2017; Morrissey & Kinderman, 2020) and lower self-rated health (Hyde, Jakub, Melchior, Van Oort, & Weyers, 2006; McKenzie, Carter, Blakely, & Ivory, 2011).

European welfare states differ in their extent of welfare provision often presented in terms of welfare regimes (Beckfield et al., 2015; Eikemo, Bambra, Joyce, & Dahl, 2008; Eikemo, Bambra, Judge, & Ringdal, 2008). Welfare regimes is an established macro determinant of public health (Bambra, 2007, 2011a, 2011b; Bambra, Netuveli, & Eikemo, 2010; Brennenstuhl, Quesnel-Vallée, & McDonough, 2012; Eikemo, Bambra, Joyce, & Dahl, 2008; Eikemo, Bambra, Judge, & Ringdal, 2008; Richter et al., 2012), that impacts health by intervening through all major social determinants of health, including access to goods and services such as education, health care, housing and social assistance (Bambra, 2011b; Beckfield & Bambra, 2016; Beckfield et al., 2015; Leão, Campos-Matos, Bambra, Russo, & Perelman, 2018). In a broader sense, welfare regimes determine conditions for social determinants (Beckfield et al., 2015) and the extent of exposure to these for different socioeconomic groups (Bambra, 2011b). However, less is known about the extent that welfare regimes modify the association between childhood economic stress (CES) and adult health. The aim of this study is two-fold: We will investigate (i) the association between childhood economic stress and adult self-rated health and cancer incidence, and (ii) if welfare regimes modify these associations. We performed secondary analyses on interactions with public social spending and income inequality to complement our welfare regime approach.

### 1.1 Welfare regimes

The work on welfare regimes has rapidly expanded since the seminal *The Three Worlds of Welfare Capitalism* by Esping-Andersen (1990). The original typology proposed three regimes (Liberal, Conservative, Social Democratic) determined by social stratification, private-public mix and de-commodification, and later de-familialization (Esping-Andersen, 1999). The typology has caused debate (Arts & Gelissen, 2002) and proposals of alternative typologies (Bambra, 2007; Eikemo, Bambra, Judge, & Ringdal, 2008). There is now largely a consensus on a five-fold typology (Eikemo, Bambra, Joyce, & Dahl, 2008) consisting of the Scandinavian (Social Democratic), Anglo-Saxon (Liberal), Bismarckian (Conservative), Southern and Eastern regime. The Anglo-Saxon regime has means-tested and targeted benefits that are often low and flat rate, combined with primarily privatized social protection (Keersbergen & Manow, 2017). The Bismarckian regime upholds existing status differentials by tying social rights to employment, with benefits set from past income, and relatively generous although conservative family benefits (Esping-Andersen, 1990). The Scandinavian regime is characterized by universal and generous benefits, a more explicit focus on social inequalities, extensive child care policies and promotion of dual-earner families (Esping-Andersen, 1990, 2009). Southern welfare states have a lack of a uniform national social assistance scheme, a labor market that is relatively segmented and favors the “happy few” in the public sector (Keersbergen & Manow, 2017), and low coverage in the health care system (Eikemo, Bambra, Joyce, & Dahl, 2008). The Eastern regime is characterized by limited health service provision and poor population health (Eikemo, Bambra, Joyce, & Dahl, 2008).

### 1.2 Welfare regimes, public health policies and population health

Welfare states affect the whole life course and may have cumulative effects on health over time, causing disparate welfare state life courses (Bambra et al., 2010). Most studies have found that the Scandinavian welfare regime provides better health compared to other welfare regimes, and this seems consistent across welfare regime typologies (Eikemo, Bambra, Judge, & Ringdal, 2008; Popham, Dibben, & Bambra, 2013; Richter et al., 2012), as well as studies on welfare state generosity, political traditions and population health (Barnish, Tørnes, & Nelson-Horne, 2018). The Scandinavian welfare regime has the highest level of individual independence of market income and family reliance (Esping-Andersen, 1990, 1999). Consequently, Scandinavian welfare states provide individuals with resources that can improve conditions throughout the life course, while sheltering from potential market and family “failures” to a more considerable extent than other welfare states (Esping-Andersen, 1999). Specific public health policies, health behavior, and healthcare systems impacts to population health (Robinson et al., 2019; Rydland et al., 2020; Thomson et al., 2018). Health behaviors (e.g. smoking, alcohol consumption, diet and physical activity) vary among European countries (Mackenbach, 2014). Overall, primary preventative (fiscal policy, workplace regulation, education) and secondary preventative measures (screening) can reduce health inequalities (Thomson et al., 2018). There is substantial variation in implementation and outcomes of primary and secondary health policies. Nordic countries have been most successful, particularly in alcohol control, child safety, and breast cancer screening, while Eastern and Southern countries have performed least well (Mackenbach & McKee, 2013). More recently, healthcare typologies have been included in comparative health research (Rydland et al., 2020). Nordic countries cluster into a performance and primary-care oriented type, and Eastern countries into a low-supply and low performance mixed type, while the remaining clusters are more mixed compared to the welfare regime typology (Reibling, Ariaans, & Wendt, 2019).

### 1.3 Life course theory on childhood conditions and adult health

From a life course perspective there are three theories on how childhood socioeconomic conditions can affect adult health (Glymour et al., 2014; Lindstrom, Hansen, & Rosvall, 2012): (i) *Embodiment* (*critical periods*) suggests that stressful and adverse experiences during sensitive developmental periods may have negative effects on later health. (ii) *Cumulative risk models* focus on how (dis-)advantages can accumulate over the life course. (iii) The *social mobility* model emphasizes socioeconomic status (SES) as an important mediator, where upwards mobility may prevent poor health. This article focuses on two health outcomes: self-rated health (SRH) and cancer. SRH is considered a global measure of health status (Wu et al., 2013) and a strong predictor of mortality (Idler & Benyamini, 1997). Studies show that CES impacts adult SRH (Lindstrom et al., 2012) and that SES is an important mediator (McKenzie et al., 2011). Adolescent and adult SRH varies across European welfare regimes, from the highest levels in Scandinavian regimes to the lower levels in Southern and Eastern regimes (Eikemo, Bambra, Judge, & Ringdal, 2008; Richter et al., 2012). There is also support for a modifying effect of welfare regimes on the association between adult SES and SRH in older-age (Sieber et al., 2019). However, there is less research on how welfare regimes may impact the association between CES and adult SRH. There is a need for more research on childhood SES and adult cancer to improve prevention efforts and population health (Vohra et al., 2016). Cancer is the second leading cause of death in Europe (OECD, 2019), with substantial social inequalities in incidence (Mihor, Tomsic, Zagar, Lokar, & Zadnik, 2020). Social factors can play a crucial role in cancer incidence as up to 40% of cancers may be attributed to lifestyle factors and environmental exposures (Colditz & Wei, 2012; Parkin, Boyd, & Walker, 2011). There is evidence for an association between lower SES in childhood and cancer incidence in adulthood, with the strongest support for stomach and lung cancer (de Kok et al., 2008; Vohra et al., 2016). There is also an increased risk of cancer onset among those with higher SES in childhood (e.g. melanoma), indicating that the relation varies by cancer site (van der Linden et al., 2018). Childhood conditions can influence adult cancer incidence (van der Linden et al., 2018) by adverse childhood experiences (“embodiment”) (Holman et al., 2016), disproportionally affecting poor households (Walsh, McCartney, Smith, & Armour, 2019); cumulative effects, e.g. by economic or educational resources that again may affect lifestyle (van der Linden et al., 2018); and through social mobility (Luo & Waite, 2005). The role of welfare states is underexamined, although cancer rates vary among countries and macroeconomic conditions may contribute (Antunes, Toporcov, & de Andrade, 2003). Few studies on CES and adult health combine data across several countries, and there is limited knowledge on how welfare regimes may alter the association between CES and adult cancer incidence. Consequently, we examine the role of welfare regimes in the association between CES and adult health.

## 2 Data and methods

### 2.1 Population and data material

The empirical analyses are based on the European Social Survey (ESS) round 7 (European Social Survey, 2018). The ESS is a biannual cross-national and cross-sectional representative survey considered among the highest quality comparative surveys in the world. The ESS employs random probability sampling of private households and collects data in face-to-face interviews. We use data from ESS Round 7 rotating module on social determinants of health which is the first pan-European survey of the general population providing reliable and harmonized data on a rich set of lifestyle and health conditions (Eikemo, Bambra, Huijts, & Fitzgerald, 2016). We include 20 European countries (*n* = 30 024), with sample sizes ranging from 981 in Slovenia to 2452 in Germany. The median response rate for the included countries was 53.3% (range: 31.4–68.9) (European Social Survey, 2015a).

### 2.2 Outcomes: Poor self-rated health and cancer

*Poor self-rated health (SRH)* is based on the variable “Subjective general health” which is a five-point ordinal count variable. “Very good”, “good”, “fair” were grouped into good SRH, and “very bad” and “bad” were grouped into poor SRH. *Cancer* is based on the variable “Have or had any health problem listed on showcard (cancer)". “Yes, currently” and “yes, previously” were grouped into cancer incidence and “no, never” into no cancer incidence.

### 2.3 Exposure: Childhood economic stress

Childhood economic stress was measured by asking how often participants and their families experienced severe financial difficulties growing up. The variable consisted of 5 categories: “Always” and “often” where coded into being exposed. “Sometimes”, “hardly ever” and “never” were coded into unexposed. Participants were provided with a text stating that "[t]he question is to be interpreted in relation to essential consumption. The family should have experienced difficulties in affording the necessities like food, clothes, housing, bills etc.” (European Social Survey, 2015b).

### 2.4 Welfare regimes

We use the welfare regime typology presented by Eikemo, Bambra, Joyce, and Dahl (2008) which expands the typology of Ferrera (1996) with the Eastern European welfare regime: Scandinavian (Denmark, Norway, Sweden, Finland), Bismarckian (Germany, France, Switzerland, Austria, Netherlands), Anglo-Saxon (United Kingdom, Ireland), Eastern (Poland, Slovenia, Czech Republic, Hungary, Estonia, Lithuania), and Southern (Spain, Portugal). Estonia and Lithuania were added to the Eastern regime based on Ebbinghaus (2012).

### 2.5 Control variables

**Fig. 1:**
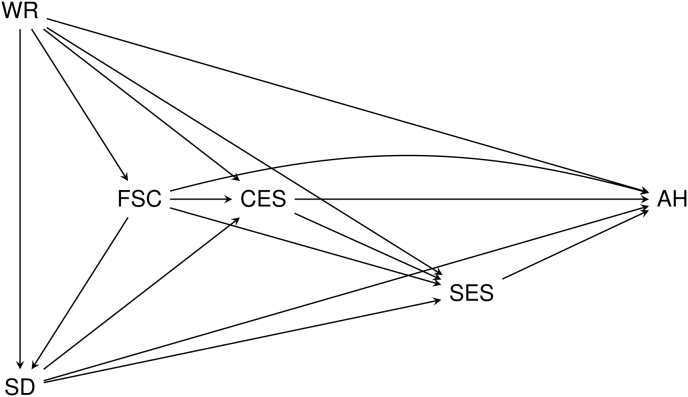
**Causal diagram for capturing factors relevant to the relationship between childhood economic stress and adult health.** The variables in the causal diagram are abbreviated as follows: Childhood economic stress (CES), adult health (AH, referring to SRH/cancer), welfare regime (WR), family socioeconomic condition (FSC), sociodemographics (SD, referring to age, gender and immigrant status) and socioeconomic status (SES).

We used a causal diagram (directed acyclic graph) to inform the choice of control variables for bias-minimized models (Elwert, 2013). We used the program DAGitty to assess our model (Textor, 2015) and shinyDAG for Fig. 1 (Creed, Aden-Buie, & Gerke, 2020). The diagram showed that the minimal set of control variables necessary to include to investigate the association between CES and SRH/cancer was welfare regime, family socioeconomic conditions, and sociodemographics (age, gender, immigrant status) (Fig. 1), where the latter is included in the diagram as a “super-node” (Tennant et al., 2019). The diagram shows that SES is an intermediate variable, and we included SES in separate models.

*Family socioeconomic condition* was measured by the father's and mother's employment situation, including information on absence/death when the participant was 14. The variable consists of 4 categories (“employed”, “self-employed”, “not working”, “dead/absent”). When both or one parent was “employed” they were grouped into “employed”, when both or one parent was “self-employed” they were grouped into “self-employed”, and when both parents were either “not working”, “absent/dead” or one not working and the other absent/dead, they were grouped into “not working, absent/dead”. Sociodemographics were measured by age, gender, and immigrant status. We included all individuals between 25 to 75 years, coded into the age cohorts “young” (25–40), “middle age” (41–59), and “late adulthood” (60–75) (Bøe et al., 2017). *Gender* was included as a binary variable coded 1 for female and 0 for male. *Immigrant status* was measured using an indicator for whether the participant was born in the country of residence (coded 0) or not (coded 1). *Socioeconomic status* was measured by the Oesch class scheme (Oesch, 2006) with four hierarchical levels and small business owners, consisting of (1) higher-grade service occupations (large employers, self-employed and employed professionals, managers), (2) lower-grade service occupations (semi-professionals and associate managers), (3) small business owners (with or without employees), (4) skilled workers (craft workers, clerks and skilled service workers) and (5) low-skilled workers.

### 2.6 Macro determinants in secondary analyses

Research suggests that public social spending (Hillier-Brown et al., 2019) and income inequality (Naik et al., 2019) are social determinants of population health. These macro determinants vary by welfare states and are included to complement our regime approach (Bergqvist, Yngwe, & Lundberg, 2013). Public social spending is a summary measure of countries' social protection systems, including redistribution and insurance against contingencies (OECD, 2019). We include OECD data on public social spending (%GDP), which consists of cash benefits, in-kind provision (goods and services), and socially oriented tax breaks that may target low-income households and economically vulnerable groups (OECD, 2020b). We also use OECD data on income inequality, measured by the Gini-coefficient of household disposable income. We use the Gini-coefficient as it is an established measure of income inequality (Jenkins & Van Kerm, 2009), often used to examine if there is a threshold beyond which effects on population health appear (Kondo et al., 2009). The Gini-coefficient contrasts the cumulative proportions of the population to the cumulative proportions of their received income (OECD, 2020a), and in our analyses ranges from 0 (perfect equality) to 100 (perfect inequality). The sample's age (median 50 years) is accounted for by using the mean value of both variables from the first year of available data (1980) to the year preceding ESS Round 7 (2013).

### 2.7 Empirical strategy

We used multilevel models to estimate the association between CES and SRH/cancer. We included a random intercept to account for between-country variation in CES and a random coefficient to account for between-country variation in the relationship between CES and SRH/cancer. As both our outcomes were prevalent, we used Poisson multilevel models to estimate risk ratios (Barros & Hirakata, 2003; Wilber & Fu, 2010). We estimated separate models for the overall association between CES and SRH, and between CES and cancer, across all 20 countries. We examined the crude association, the association adjusted for the minimal set, and finally when adjusted for the minimal set and SES. We estimated separate models with an interaction between CES and welfare regimes for SRH and cancer. We assessed the interaction by estimating predicted probabilities for SRH and cancer for exposed to CES across welfare regimes. We hold the minimal set at mean value to obtain the predicted probability of the outcome for the average person exposed to CES (Williams, 2012). We used the Scandinavian regime as reference category and assessed differences among other regimes by changing reference category. We estimate separate models to examine if the predicted probability of poor SRH/cancer among exposed to CES differs between those with lower FSC (parents unemployed/absent) and higher FSC (parents employed) in different welfare regimes, with the minimal set held at mean value. In our secondary analyses, we examined interactions between CES and public social spending and income inequality in separate models. We compared the difference in predicted probability of poor SRH and cancer between exposed and unexposed to CES over values of public social spending and income inequality holding the minimal set at mean value. Analyses were conducted on complete cases with 95% (minimal set) and 92% (minimal set with SES) for SRH, and 89% (minimal set) and 86% (minimal set with SES) for cancer. Weights were applied according to ESS guidelines (European Social Survey, 2014). All statistical analyses were conducted in Stata SE 16.1. (StataCorp, 2020).

## 3 Results

### 3.1 Childhood economic stress and adult health

Overall, 16% (*n* = 4083) was exposed to CES, with the highest prevalence in the Anglo-Saxon regime and the lowest prevalence in the Scandinavian regime. The distributions of participants characteristics are presented by exposure to CES in Table 1. Participants exposed to CES had higher prevalence of poor SRH and cancer relative to unexposed in all welfare regimes. The proportion of poor SRH was highest in the Eastern and Southern regime, and lowest in the Scandinavian and Anglo-Saxon regime, while the proportion of cancer was highest in the Eastern and Anglo-Saxon regime, and lowest in the Southern regime. Participants exposed to CES were less likely to be in higher-grade service occupations, and more likely to be immigrants, females, and have parents that were not working or absent/dead. The Scandinavian regime had the highest amount of public social spending and the lowest amount of income inequality.

**Table 1:** Participant characteristics by welfare regime and childhood economic stress.

	Scandinavian (*n* = 5326)		Bismarckian (*n* = 9559)		Anglo-Saxon (*n* = 3810)		East European (*n* = 8833)		South European (*n* = 2496)	
Childhood economic stress	Yes, *n* (%)	No, *n* (%)	Yes, *n* (%)	No, *n* (%)	Yes, *n* (%)	No, *n* (%)	Yes, n (%)	No, *n* (%)	Yes, *n* (%)	No, *n* (%)
	632 (12)	4656 (88)	1264 (15)	8173 (85)	741 (19)	1927 (81)	1734 (18)	6847 (82)	432 (15)	2057 (85)
Self-rated health										
Poor	266 (41)	1059 (22)	544 (46)	2224 (32)	273 (41)	594 (23)	1080 (55)	2632 (36)	262 (54)	794 (36)
Good	366 (59)	3595 (78)	719 (54)	5947 (68)	468 (59)	2403 (77)	649 (45)	4204 (64)	170 (46)	1262 (64)
Cancer										
Currently or previously	63 (10)	445 (10)	160 (10)	810 (9)	113 (15)	264 (8)	310 (16)	680 (11)	45 (9)	133 (6)
Never	566 (90)	4201 (90)	1097 (90)	7334 (91)	616 (85)	2706 (92)	1120 (84)	4596 (89)	382 (91)	1917 (94)
Family socioeconomic condition										
Employed	476 (79)	3708 (81)	875 (73)	6388 (80)	503 (81)	2167 (82)	1356 (76)	5925 (80)	284 (65)	1439 (72)
Self-employed	105 (15)	813 (17)	182 (14)	1374 (16)	99 (6)	567 (13)	110 (18)	432 (17)	98 (26)	500 (25)
Not working or absent/dead	33 (6)	78 (2)	139 (13)	264 (4)	99 (14)	159 (5)	148 (6)	232 (3)	35 (8)	71 (3)
Gender										
Female	358 (57)	2251 (48)	691 (56)	4120 (50)	405 (58)	1638 (53)	1053 (58)	3857 (55)	235 (50)	1017 (48)
Male	274 (43)	2405 (52)	573 (44)	4053 (50)	336 (42)	1350 (47)	675 (42)	2984 (45)	197 (50)	1040 (52)
Immigrant status										
Yes (moved to country)	549 (84)	4229 (90)	957 (80)	7167 (88)	620 (88)	2573 (83)	1595 (97)	6465 (98)	391 (87)	1874 (92)
No (born in country)	83 (16)	427 (10)	307 (20)	1006 (12)	121 (12)	423 (17)	138 (3)	379 (2)	41 (13)	183 (8)
Socioeconomic status										
Higher grade service occupations	95 (16)	1174 (25)	157 (11)	1690 (21)	72 (14)	566 (21)	163 (10)	1104 (15)	26 (7)	306 (15)
Lower grade service occupations	129 (21)	1104 (25)	222 (19)	1765 (23)	113 (18)	467 (19)	236 (14)	1159 (18)	36 (11)	236 (15)
Small business	60 (9)	441 (9)	128 (11)	831 (10)	96 (12)	397 (14)	131 (12)	725 (15)	65 (18)	272 (15)
Skilled worker	221 (34)	1307 (28)	403 (34)	2474 (32)	213 (28)	890 (29)	609 (36)	2312 (34)	128 (29)	619 (30)
Unskilled worker	120 (20)	578 (13)	308 (25)	1189 (15)	196 (28)	519 (17)	520 (28)	1296 (18)	145 (35)	475 (24)
Age (mean)	52	50	51	49	53	49	53	48	53	48
Gini (0–100)	24.7		28.8		33.4		29.9		35.5	
Public social spending	24.4		23		17.7		19		15.3	

Note: Data are weighted. Rounded percentages are shown.

Exposure to CES was associated with an increased risk of poor SRH (crude risk ratio (RR) 1.53, CI 95% 1.42–1.66) and cancer (crude RR 1.38, CI 95% 1.26–1.52). Adjustment for the minimal set slightly reduced the risk for poor SRH (RR 1.41 CI 95% 1.29–1.54) and cancer (RR 1.19, CI 95% 1.02–1.37). Adjustment for SES produced reduced risk for poor SRH (RR 1.36, CI 95% 1.25–1.48), and similar estimates for cancer (RR 1.18, CI 95% 1.02–1.38) (see Table 2). We found support for a difference in predicted probability of poor health among exposed to CES between those with lower and higher FSC in all welfare regimes, with the smallest difference in the Scandinavian (- 3%, CI 95% - 6 to −0.01, *p* = 0.043) and the largest in the Southern (- 5%, CI 95% −9.8 to −0.03, *p* = 0.039) and Eastern regime (−4.9%, CI 95% −9.6 to −0.02, *p* = 0.039). We did not find support for differences for cancer.

**Table 2:** Multilevel models for association between childhood economic stress, poor self-rated health and cancer.

Model		(1)	(2)	(3)	(4)	(5)	(6)
Variables	Categories	Unadjusted	Minimal set	Minimal set + SES	Unadjusted	Minimal set	Minimal set + SES
Outcome		Poor SRH	Poor SRH	Poor SRH	Cancer	Cancer	Cancer
		RR (95% CI) *p*-value	RR (95% CI) *p*-value	RR (95% CI) *p*-value	RR (95% CI) *p*-value	RR (95% CI) *p*-value	RR (95% CI) *p*-value
Childhood economic stress		1.53 (1.42–1.66)	1.41 (1.29–1.54)	1.36 (1.25–1.48)	1.38 (1.26–1.52)	1.19 (1.02–1.37)	1.18 (1.02–1.38)
		< 0.000	< 0.000	< 0.000	< 0.000	0.023	0.032
Welfare regime	Scandinavian		(ref.)	(ref.)		(ref.)	(ref.)
	Bismarckian		1.38 (1.10–1.72)	1.34 (1.08–1.67)		0.93 (0.78–1.12)	0.93 (0.78–1.11)
			0.005	0.009		0.46	0.43
	Anglo-Saxon		1.06 (0.91–1.25)	1.02 (0.87–1.20)		0.91 (0.78–1.06)	0.92 (0.79–1.07)
			0.45	0.78		0.23	0.26
	East European		1.66 (1.41–1.96)	1.57 (1.34–1.84)		1.10 (0.78–1.55)	1.08 (0.76–1.54)
			< 0.000	< 0.000		0.59	0.65
	South European		1.72 (1.41–2.09)	1.60 (1.33–1.93)		0.67 (0.56–0.81)	0.69 (0.58–0.82)
			< 0.000	< 0.000		< 0.000	< 0.000
Family socioeconomic condition	Not working or absent/dead		(ref.)	(ref.)		(ref.)	(ref.)
	Self-employed		0.81 (0.72–0.91)	0.85 (0.77–0.93)		0.85 (0.58–1.24)	0.91 (0.64–1.31)
			< 0.00	< 0.00		0.40	0.63
	Employed		0.89 (0.82–0.96)	0.92 (0.85–1.00)		0.85 (0.58–1.26)	0.90 (0.62–1.30)
			0.002	0.041		0.42	0.56
Female			1.15 (1.09,1.22)	1.11 (1.04–1.18)		1.35 (1.22,1.50)	1.36 (1.22–1.50)
			< 0.000	0.0014		< 0.000	< 0.000
Immigrant status			0.99 (0.90–1.08)	1.01 (0.91–1.13)		0.98 (0.88–1.09)	0.96 (0.85–1.08)
			0.78	0.79		0.75	0.47
Age group	Young (25–40)		(ref.)	(ref.)		(ref.)	(ref.)
	Middle age (41–59)		1.83 (1.57–2.13)	1.82 (1.58–2.08)		2.54 (2.23–2.89)	2.54 (2.23–2.89)
			< 0.000	< 0.000		< 0.000	< 0.000
	Late adulthood (60–75)		2.43 (1.97–2.99)	2.39 (1.95–2.92)		4.90 (4.20–5.71)	4.78 (4.08–5.61)
			< 0.000	< 0.000		< 0.000	< 0.000
Socioeconomic status	Higher-grade service occupations			(ref.)			(ref.)
	Lower-grade service occupations			1.26 (1.18–1.34)			1.07 (0.90–1.28)
				< 0.000			0.45
	Small business owner			1.29 (1.18–1.42)			0.96 (0.85–1.07)
				< 0.000			0.46
	Skilled workers			1.50 (1.42–1.59)			1.04 (0.92–1.18)
				< 0.000			0.55
	Unskilled workers			1.74 (1.64–1.85)			1.06 (0.93–1.19)
				< 0.000			0.39
Statistics	*N*	29511	28625	27719	27558	26776	25949

### 3.2 Interaction analyses

**Fig. 2:**
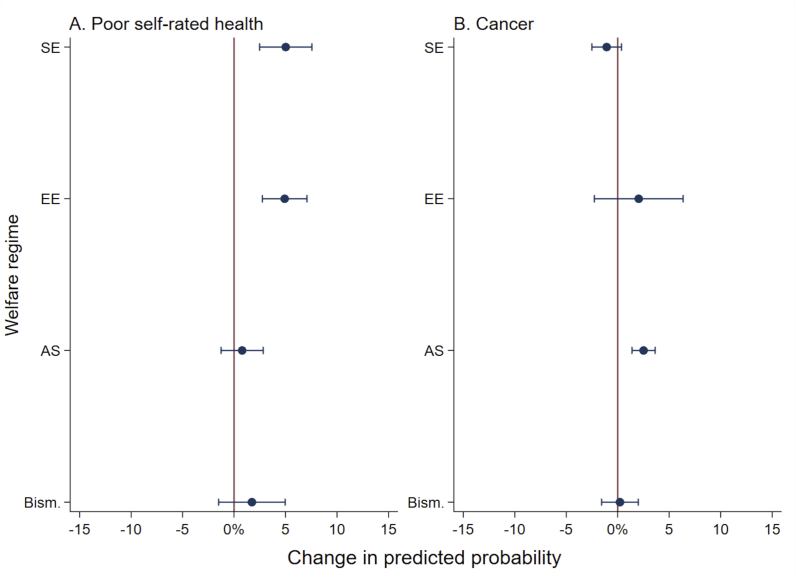
**Change in predicted probability of poor self-rated health and cancer among exposed to childhood economic stress in welfare regimes relative to the Scandinavian welfare regime.** Welfare regime abbreviations: Bismarckian (Bism.), Anglo-Saxon (AS), East European (EE), South European (SE). Line at zero on *x*-axis indicate no change.

There was support for an interaction between welfare regime and CES for poor SRH and cancer for the average person exposed to CES (Fig. 2). From Fig. 2 (A) there is evidence for an increased predicted probability of poor SRH among exposed to CES in the Southern (5%, CI 95% 2.5–7.6) and Eastern regime (4.9%, CI 95% 2.8–7.1), relative to the Scandinavian regime. There is also support for an increased predicted probability of cancer among exposed to CES in the Anglo-Saxon (2.5%, CI 95% 1.4–3.6**,** *p* < 0.000) relative to the Scandinavian regime (Fig. 2B).

Compared to the Bismarckian regime, there was a 3.2% (CI 95% 0.3–6.1, *p* = 0.031) increase in predicted probability of poor SRH in the Eastern regime and a 3.3% (CI 95% 0.1–6.5, *p* = 0.045) increase in the Southern regime. There was also a 4.1% (CI 95% 2.5–5.8, *p* < 0.000) increase in predicted probability of poor SRH in the Eastern regime, and a 4.2% (CI 95% 2.2–6.3, *p* < 0.000) increase in the Southern regime, relative to the Anglo-Saxon regime. For cancer, there was a 2.3% (CI 95% 1–3.6, *p* < 0.000) increase in predicted probability for cancer in the Anglo-Saxon regime, relative to the Bismarckian regime. There was a 3.6% (CI 95% 2.6–4.5, *p* < 0.000) increase in probability of cancer in the Anglo-Saxon regime, relative to the Southern regime.

### 3.3 Secondary analyses

**Fig. 3:**
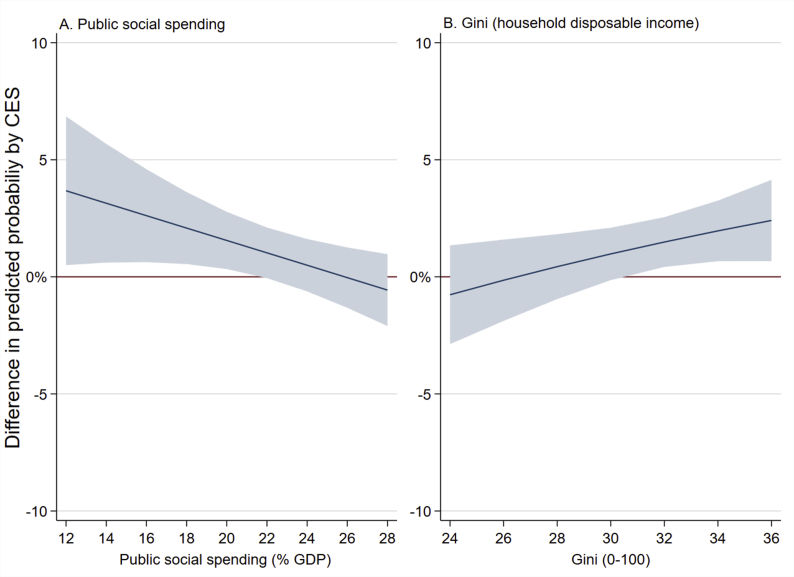
Change in difference of predicted probability of poor self-rated health and cancer between exposed and unexposed to childhood economic stress over values of public social spending and income inequality. Line at zero on *y*-axis indicates no change.

Results from the secondary analyses are presented in Fig. 3. There was support for an interaction between CES and Gini-coefficient (*p* = 0.03) and public social spending (*p* = 0.019) for cancer, but not poor SRH. For public social spending, there was support for a declining difference in the predicted probability of cancer between exposed and unexposed from 3.7% (CI 95% 0.05–6.9) at a public social spending value of 12 to 1.6% (CI 95% 0.03–2.8) at a value of 12, and no support for a difference between value over this. For Gini, there was no support for a difference in probability of cancer up to a Gini value of 30, but evidence for an increasing divergence from 1.5% (CI 95% 0.04–2.6) at 32 to 2.4% (CI 95% 0.07–4.1) at 36.

## 4 Discussion

In this large comparative study of 30 024 individuals in 20 European countries, we assessed the association between childhood economic stress (CES) and poor self-rated health (SRH) and cancer incidence (any type), and how welfare regimes may modify these associations. We conducted secondary analyses on the modifying effect of public social spending and income inequality. We have three main findings.

First, CES was associated with an increased risk of poor SRH and cancer incidence. Our findings are consistent with studies on childhood socioeconomic conditions and adult SRH (Lindstrom et al., 2012; McKenzie et al., 2011; Niedzwiedz, Pell, & Mitchell, 2015), and cancer incidence (de Kok et al., 2008; Vohra et al., 2016). To our knowledge, there are few studies on childhood socioeconomic conditions and cancer combining several countries, and no studies accounting for the role of welfare regimes. Most studies on childhood socioeconomic conditions and cancer use father's SES and mortality data. Our approach focus on CES which is likely to capture financial hardship that may have independent effects on health (Conklin et al., 2013), and show that this approach provide similar results. Our results can be interpreted in line with all the three proposed models from life course theory (see 1.3.), as exposure to CES may occur in critical periods, initiate accumulation of disadvantage, and impact adult SES. Particularly, our results provide evidence for the social mobility model for SRH as the risk of poor SRH decreased by controlling for SES. However, the risk of cancer does not decrease by controlling for SES, in line with existing research (de Kok et al., 2008; van der Linden et al., 2018). This may be related to SES-specific health behaviors, e.g. lower SES is associated with smoking and lung cancer (Vohra et al., 2016), while higher SES may increase skin cancers by UV exposure through holidays abroad (Shack, Jordan, Thomson, Mak, & Moller, 2008; van der Linden et al., 2018).

Second, there was an increased probability of poor SRH in the Southern and Eastern regime, and increased probability of cancer in the Anglo-Saxon regime among exposed to CES, relative to the Scandinavian regime. Despite not finding support for a difference with other regimes, it is relevant to emphasize that absence of evidence is not evidence of absence (Altman & Bland, 1995). These findings show that the more comprehensive Scandinavian welfare regime alleviates negative consequences of early economic stress on adult SRH and cancer. While there are few studies examining specifically how the association of early economic conditions may affect adult SRH, these findings are consistent with evidence showing that socioeconomic conditions over the life course have weaker associations with well-being in Scandinavian compared to other European regimes (Niedzwiedz et al., 2015). The Scandinavian regime also had the smallest difference in probability of poor health between exposed to CES from lower and higher socioeconomic backgrounds, indicating that family background may be more important for adult health in less comprehensive welfare states. As welfare regimes intervene through many social determinants of health, there may be several plausible mechanisms at play. One important mechanism may go through the Scandinavian regime's more explicit focus on promoting equal opportunities compared to other European regimes. The relative more extensive welfare provision that impacts family benefits, social protection, education and health care, as well as effective health policies (Mackenbach & McKee, 2013), contributes to higher social mobility rates and effective poverty reduction in Nordic countries (Fouarge & Layte, 2005; OECD, 2018). Consequently, when combining the welfare regime and life course perspectives, Scandinavian welfare regimes may mitigate negative impacts of adverse exposures during critical periods, alleviate accumulation processes through several of the channels mentioned above, and through promoting equal opportunities.

Third, increases in public social spending decreased the gap in the probability of cancer among exposed and unexposed to CES, while increases in income inequality increased these differences. The secondary analyses provide supporting evidence for our analyses using the welfare typology, as the Scandinavian regime has the highest rate of public social spending and the lowest level of inequality. Studies have found that public social spending is associated with mortality and life expectancy (Bergqvist et al., 2013). We do not know of any studies examining the interaction between CES and cancer by public social spending. In line with research on income inequality and health, we find support for the threshold effect hypothesis which states the there is a threshold beyond which income inequality starts impacting health (Kondo et al., 2009).

## 5 Strengths and limitations

The main strength of our study is the multilevel design used on a large high-quality data set combined with the use of a causal diagram to inform the selection of control variables. We have two outcome measures allowing use to assess how welfare regimes may alter the relationship between SRH as a global health measure, and cancer incidence as a more specific disease. We draw on an interdisciplinary framework combining welfare regime and epidemiological life course theory. Despite the study being reliant on cross-sectional data, the temporal order of CES and adult health is unlikely an issue. This study is subject to the following limitations. Some limitations are due to attributes of the ESS data. The median response rate for the included studies was 53.3%. The validity of SRH in cross-national comparisons has been questioned (Jylha, Guralnik, Ferrucci, Jokela, & Heikkinen, 1998). Self-reported cancer may be affected by false-negative reporting bias (Desai, Bruce, Desai, & Druss, 2001). Cancer represents a heterogeneous group of diseases, and we were not able to account for how the role of CES may vary by cancer type (de Kok et al., 2008). Our measure of CES is subjective, and may alongside FSC be subject to recall bias (Bøe et al., 2017). Parental occupation or education could provide an alternative measure to FSC, but there was considerable missing data in both variables. Moreover, income is arguably the most important mitigating factor for CES, which is captured by employment information in FSC. Finally, longitudinal research designs are needed to provide more information on causal relationships, as well as which specific dimensions of welfare regimes that are most effective in reducing the negative health effects of CES.

## 6 Conclusion

Childhood economic stress is associated with increased risk of poor self-reported health and cancer incidence across 20 European countries and welfare regimes modify these associations. Those exposed to CES were more likely to have poor SRH in the Southern and Eastern regime, and cancer in the Anglo-Saxon regime, relative to the Scandinavian regime. These findings emphasize that welfare regimes are important macro determinants of how CES affects adult SRH and cancer. Longitudinal research is needed to increase understanding of specific mechanisms, welfare state policies, and how the timing of social exposures may affect the association between CES and adult SRH and cancer incidence.

## CRediT authorship contribution statement

**Tarjei Widding-Havneraas:** Conceptualization, methodology, investigation, writing – original draft. **Siri Hansen Pedersen:** Conceptualization, methodology, writing – original draft.

## Ethical statement

This study is based on European Social Survey (ESS) data. More information about research ethics in ESS can be found at https://www.europeansocialsurvey.org/about/ethics.html.

## Funding

The study received no funding.

## Declaration of competing interest

The authors declare that there are no potential conflicts of interest with respect to the research, authorship, or publication of this article.
